# Enhancing Surgical Outcomes: A Critical Review of Antibiotic Prophylaxis in Orthopedic Surgery

**DOI:** 10.7759/cureus.47828

**Published:** 2023-10-27

**Authors:** Gaurav K Upadhyyaya, Sachchidanand Tewari

**Affiliations:** 1 Department of Orthopedics, All India Institute of Medical Sciences, Raebareli, Raebareli, IND; 2 Department of Pharmacology, All India Institute of Medical Sciences, Raebareli, Raebareli, IND

**Keywords:** death, centers for disease control and prevention, antibiotic resistance, clinical outcomes, economic efficiency, extended hospitalizations, antibiotic prophylaxis, surgical site infections

## Abstract

The postoperative burden remains significant due to the possibility of prolonged hospitalization, escalated healthcare costs, and patient distress caused by postorthopedic surgical site infections (SSIs). Orthopedic surgery is likewise faced with a significant challenge posed by these conditions. A positive association has been observed between the presence of postorthopedic SSIs and heightened susceptibility to adverse health outcomes, along with elevated rates of morbidity and mortality. Systemic antibiotic prophylaxis (SAP) reduces the risk of acquiring an SSI. Closed fractures, open fractures, arthroplasty, and percutaneous fixation each possess distinct attributes that impact the data and antimicrobial therapy. When implementing SAP, it is crucial to strike a delicate equilibrium between maintaining effective antibiotic stewardship protocols and preventing the occurrence of SSIs. This practice effectively prevents both the incidence of negative consequences and the emergence of antibiotic resistance. The objective of this study was to examine the existing literature on the use of surgical antibiotic prophylaxis in orthopedic surgery and explore the potential consequences associated with the inappropriate administration of antibiotics.

## Introduction and background

In the realm of modern medicine, orthopedic surgery is a testament to the extraordinary breakthroughs that have been made in enhancing the well-being of a large number of people [1]. Orthopedic surgery is a crucial component in the effort to improve musculoskeletal health [1-3]. Orthopedic surgery plays a crucial role in addressing many musculoskeletal conditions, such as persistent pain through joint replacement therapy or restoring mobility through fracture repair. However, despite the significant advancements in surgical techniques, the issue of surgical site infections (SSIs) continues to pose a considerable and complex concern [2].

While orthopedic surgery has intrinsic good qualities, it is important to carefully evaluate potential negative effects. The emergence of SSIs, which can lead to extended hospital stays, heightened healthcare costs, patient distress, and, in some cases, significant morbidity [3,4], is a highly worrisome issue. The utilization of antibiotic prophylaxis has become a crucial element in the perioperative treatment of orthopedic patients [4]. This practice is implemented with the purpose of reducing the potential risks associated with the infection to prevent future complications.

Antibiotic prophylaxis is an intentional practice of administering antibiotics prior to surgical treatments, with the aim of mitigating the risk of infection, particularly at the surgical site [5]. This measure is implemented with the aim of preventing the dissemination of the SSI to other anatomical regions. The utilization of prophylactic antibiotics is well recognized as an effective measure in reducing the incidence of bloodstream SSIs. However, the specific methods and practices linked to its administration are subject to ongoing development and enhancement [6]. Medical personnel are frequently confronted with significant inquiries in their pursuit of optimal surgical outcomes for orthopedic patients when it comes to the selection of antibiotics that are likely to be efficacious [7]. When would be the best time for the administration to have this discussion? In view of the rising worry over antibiotic resistance, it has become important to investigate the most effective strategy for striking a balance between the possible benefits connected with the development of antibiotic resistance and the advantages that are linked with prophylaxis.

This comprehensive study aims to give a comprehensive review of antibiotic prophylaxis in the context of orthopedic surgery. This crucial component of perioperative care is the focus of this research, and its purpose is to undertake a complete review of the current body of information, evaluate recent advancements, and give insights into the numerous complicated aspects that impact this essential aspect of perioperative care. We are about to begin an in-depth investigation of the use of antibiotic prophylaxis in the context of orthopedic surgery. Meanwhile, we would like to extend an invitation to readers to investigate the existing body of research, ongoing debates, and emerging tendencies in this field. The shared goal of enhancing the safety and efficacy of orthopedic therapies is what motivates this pursuit of excellence.

## Review

Reasons for the need of antibiotic prophylaxis in orthopedic surgery

Antibiotic prophylaxis, often known as prophylactic antibiotics, is the practice of giving antibiotics to patients before a medical operation or other potentially infectious occurrences [8-15]. Antibiotic prophylaxis is also widely referred to as prophylactic antibiotics. It's critical to distinguish between using antibiotics to treat an existing infection and using them to prevent future illnesses. Prophylactic antibiotic treatment is administered when there is a high risk of acquiring a bacterial infection; as a result, the number of infections that do take place is reduced [9]. When antibiotics are given to a patient before they get therapy or are exposed to a potentially infectious agent, their primary function is to reduce or eliminate any germs that may already be present in the body [10].

Antibiotic Precautions in Typical Cases

It is occasionally advised to take antibiotics before surgery to lower the risk of SSIs. The likelihood of bacterial infection is particularly high following abdominal procedures, knee replacements, and heart valve replacements [11]. In addition, it is also common in joint replacements. Before dental procedures, which might introduce bacteria into the circulation, antibiotics are occasionally provided to individuals who have been diagnosed with heart disease or who have artificial joints [12]. Endocarditis, which is an infection of the lining of the heart, and joint infection caused by bacteria are both preventable. Patients with compromised immune systems who are receiving therapies such as chemotherapy or organ transplantation are sometimes given prophylactic antibiotics to avoid opportunistic infections [8-12]. This is done in an effort to protect the patient from potentially life-threatening illnesses. Before departing for high-risk locations (such as those where malaria is a prevalent disease), tourists may decide to start a course of antibiotics at home as a preventative measure.

Factors to Think About

Isolated antibiotics: When choosing antibiotics for prophylactic measures, it is important to take into account the bacterial strains that are most likely to be present during the treatment or event in question. This helps ensure that the appropriate medication is administered. Antibiotics that are capable of killing particular germs are what are going to be used [13]. Prophylactic antibiotic treatment typically consists of a single dosage taken either before undergoing surgery or before being exposed to possibly pathogenic germs. To treat an illness with an antibiotic at a dosage that is enough high to kill or suppress any bacteria present at that moment, but not to treat the infection permanently [14], the objective is to kill or suppress any bacteria that are now present.

The moment: Time will tell how things turn out. The drug is typically given right before surgery [16-20] so that appropriate antibiotic levels are accessible in the body during the potentially dangerous window before surgery or exposure. This helps to guarantee that adequate levels of antibiotics are available in the body. When determining which antibiotic to use for prophylaxis, physicians should take into account a patient's history of allergies or sensitivities to the antibiotics in question. Prophylactic antibiotic treatment is used when there is a greater potential for infection than there is for undesirable outcomes caused by the medication, such as allergic reactions, unpleasant effects, or antibiotic resistance [15]. In conclusion, antibiotic prophylaxis refers to the practice of administering antibiotics in carefully chosen clinical situations in order to avoid the spread of illness. This time-honored medical procedure is governed by clinical rules, which ensure the patients' well-being while simultaneously encouraging the most beneficial outcomes for their health.

Importance of SSI Prevention 

The reduction of the risk of SSIs in the medical field is essential for a variety of reasons [1-5].

Patient well-being and safety: The health and safety of the patient is the first priority. In extreme circumstances, SSI can result in considerable difficulties, including extended hospital stays, higher expenditures associated with medical treatment, and even death [17]. Putting patient safety first by avoiding SSIs.

Reduced costs of healthcare: The treatment for SSI can be expensive. When they are avoided, patients and the healthcare system are relieved of the additional financial load that they cause [18]. This may involve extra stays in the hospital, the administration of drugs, modifications to the original surgical procedure, and a prolonged period of recuperation.

Decreased lengths of stay in hospital: SSI usually leads to increased lengths of stay in the hospital, which can cause major disruptions in a patient's life and raise the risk of various issues linked with healthcare [19]. The reduction in lengths of stay in hospitals caused by the avoidance of SSI makes available more beds for other patients. The number of staff members, pieces of equipment, and beds that are available in hospitals and other healthcare institutions is sometimes restricted. These resources will be freed up to be given to patients who are in need of care if SSIs are avoided. Patients who are recovering from SSIs may feel discomfort, anguish, and a decrease in their quality of life. Patients who had a higher quality of life before may not. Patients have a better chance of having an easier and less painful recovery if they take steps to prevent SSIs.

The predominant approach for managing SSIs entails the administration of antibiotics. However, it is important to note that excessive or inappropriate utilization of antibiotics might contribute to the emergence of antimicrobial-resistant bacteria. The issue of antibiotic resistance is a global concern, and one potential strategy to address this problem is to decrease the frequency of antibiotic usage for the treatment of infections occurring at surgical sites.

Increases in reliability and confidence: Healthcare facilities that can avoid SSIs on a continuous basis earn a reputation for delivering excellent treatment [20]. Patients are more inclined to pick hospitals with lower SSI rates and trust such institutions, which can result in an increase in revenue for healthcare providers. Patients are more likely to choose and trust hospitals with lower SSI rates.

Considering the law and our values: It is the ethical and moral responsibility of those who work in the healthcare industry to offer treatment that is both safe and effective [4]. If proper safety measures are not taken to prevent SSIs, the failure to do so may result in legal and ethical ramifications, including allegations of malpractice.

Investigation and experimentation: It is important to prevent SSIs because doing so encourages research and development in the healthcare industry, which in turn leads to advancements in surgical procedures, infection control, and the development of new technology and products that are intended to prevent SSIs [6-9].

Concerning the public: The effects of SSI can be felt on the public's health [10-14]. Infections that spread in hospital settings can have repercussions not just for the individuals who contract them but also for the community. The improvement of public health results from the prevention of SSIs [15-20].

In conclusion, the prevention of SSIs is critical for the health of patients, the continued economic sustainability of healthcare systems, and the overall improvement of the quality of healthcare. This involves maintaining proper cleanliness, adhering strictly to infection control policies, providing appropriate preoperative and postoperative care, and ensuring that medical workers get ongoing education and training.

Current guidelines and recommendations

These recommendations aim to reduce the incidence of SSIs by recommending the use of antibiotics both before and after orthopedic procedures. Given that policies may be susceptible to change over time, it is important to review these organizations' most current changes. We'll go through a summary of the ground rules for a few illustrious professional organizations in the sections that follow.

American Academy of Orthopedic Surgeons

The guidelines for antibiotic prophylaxis in orthopedic surgery are provided by the American Academy of Orthopedic Surgeons (AAOS). They highlighted how important it is to provide prophylactic antibiotics one hour before making the incision for the surgical procedure. The type of surgery being performed, the prevalent bacterial resistance patterns in the area, and any known patient sensitivities all factor into the decision of which antibiotics to prescribe. Cefazolin and vancomycin are two antibiotics that are frequently used when specific conditions are present. In most cases, prophylaxis is given no later than 24 hours following surgery, unless there are exceptional conditions that require it to be given earlier [21].

Infectious Diseases Society of America

In the context of surgical procedures, especially orthopedic operations, the Infectious Diseases Society of America (IDSA) provides guidelines for the use of antibiotic prophylaxis. The recommendations take into account a number of different criteria, among which include the particular surgical operation, the patient's medical history, and regional trends in bacterial resistance. Cefazolin is the antibiotic of choice for the majority of surgical procedures involving the musculoskeletal system [22].

Surgical Care Improvement Project 

The Surgical Care Improvement Project (SCIP) is a national project that aims to reduce postoperative complications such as SSIs. It details the regimen and choice of antibiotics that should be used during surgical operations, including orthopedics. The SCIP suggests that prophylactic antibiotics be given either within two hours after the surgical incision in the case of vancomycin and fluoroquinolones or within one hour in the case of other antibiotics. The selection of antibiotics must be appropriate for the surgical treatment, and the duration of prophylaxis should not go beyond 24 hours [23].

Centers for Disease Control and Prevention 

The Centers for Disease Control and Prevention (CDC) provides guidelines for reducing the risk of SSIs across a range of medical settings and procedures. Although these recommendations do not focus on orthopedic surgery in particular, the advice they offer for avoiding infections is invaluable. Recommendations about the appropriate time of prophylactic antibiotics and factors to consider before stopping antibiotic treatment after surgical procedures are among the most important [24].

It is critical to keep in mind that these recommendations might be revised in light of newly discovered information and altered patterns of antibiotic resistance. In addition, exact suggestions may change depending not just on the kind of orthopedic operation being performed but also on the unique qualities of the patient.

Discussion of recommended antibiotics and their dosages on antibiotic prophylaxis in orthopedic surgery

Antibiotic prophylaxis is crucial in reducing the risk of infection during orthopedic surgery. The type of procedure to be performed, the patient's individual circumstances, and the regional incidence of antibiotic resistance should all be considered when deciding which antibiotics to use and how much to deliver. Careful administration of antibiotics is required not just to prevent superbug infections but also to lessen the likelihood of antibiotic resistance and adverse consequences.

List of Popular Antibiotics and Their Dosages for Various Orthopedic Surgeries

Cefazolin: Cefazolin [25] is the antibiotic that is utilized for orthopedic prophylaxis the majority of the time. *Staphylococcus aureus*, one of the germs most likely to be responsible for SSIs, is well defended against by this substance. The typical dosage consisted of a single dose of 1-2 g given by intravenous administration within 30 minutes following the surgical incision. If the surgery is going to take a long time, you might want to give the patient an extra dosage every four hours.

Clindamycin: Clindamycin [26] is an excellent option for those who cannot take penicillin or cephalosporin antibiotics because they are allergic to them. Within the first half an hour after the surgery, an intravenous dose of 600-900 mg is often given.

Vancomycin: Those with known or suspected infections caused by methicillin-resistant *Staphylococcus aureus* (MRSA) or those who are at high risk for MRSA should not take vancomycin [27]. In most cases, the dose of 15 mg/kg is given intravenously one to two hours before the procedure.

Aminoglycosides like gentamicin: Cefazolin may be used with aminoglycosides like gentamicin in some surgical procedures, such as total joint arthroplasty [28]. In most cases, the intravenous dose is between 1 and 2 mg/kg, and it is given 30 minutes before the incision.

Cefuroxime: This medication may be used for patients undergoing operations that impact the lower extremities or for situations in which more gram-negative coverage is necessary [29]. The normal dose was 1.5 g administered intravenously 30 minutes before surgery.

Ciprofloxacin: Ciprofloxacin may be used in certain situations, such as when there is a high risk of infection with gram-negative germs [30]. When there is a significant chance of infection with gram-positive germs, ciprofloxacin may also be taken. Usually, 400 mg is the intravenous dosage.

Prior to starting the surgical incision, it is essential to highlight the quick delivery of prophylactic antibiotics. Antibiotic concentrations in tissue at the moment of incision are most effective if they are administered at the optimal time [30,31]. Antibiotic prophylaxis should generally be limited to the intraoperative and early postoperative periods, lasting fewer than 24 hours, to lessen the likelihood of bacteria developing resistance to antibiotics and experiencing adverse effects [29,30].

Guidance and recommendations may vary depending on criteria such as the nature of the orthopedic procedure being performed, the patient's individual risk factors, and the prevalence of antibiotic resistance in the area. As a result, orthopedic surgeons should consult infectious disease specialists for guidance or follow the guidelines established by their institutions to guarantee that the antibiotics selected and doses administered are suitable for each patient.

Importance of timing and duration of antibiotic prophylaxis in orthopedic surgery 

Antibiotic prophylaxis, namely its timing and duration, is crucial in avoiding SSIs. Antibiotic prophylaxis is when antibiotics are given to a patient before surgery in order to protect them from getting an infection [32]. Antibiotic prophylaxis is highly effective in preventing the development of antibiotic resistance if it is administered at the appropriate time and for the appropriate amount of time. In orthopedic surgery, the following are the most significant considerations to consider in terms of whether antibiotic prophylaxis should be used and for how long:

Timing of Preoperative Events

Antibiotics have to be given to the patient before the incision is made during surgery, ideally within 30-60 minutes. This scheduling guarantees that a suitable concentration of the medicine will be present in the bloodstream throughout the surgical process. If antibiotics are administered too early, this might result in medication levels that are less than ideal during surgery; nevertheless, if they are administered too late, this could result in inadequate protection [33-35].

Intraoperative Dosing

It may be necessary to administer extra antibiotic doses in order to maintain effective medication levels after some orthopedic operations, particularly those that last for an extended period of time or involve a considerable amount of blood loss. These intraoperative dosages must be administered in a way that considers both the antibiotic's pharmacokinetics and the length of the procedure [36].

Postsurgical Dosing

If the operation takes a long time or there is a significant possibility of infection, a postoperative dosage can be required. On the other hand, postoperative supplementation is not suggested following the majority of orthopedic surgeries. Extended prophylaxis is typically discouraged as a practice since doing so can lead to unnecessary antibiotic exposure as well as the development of antibiotic resistance [37].

The Duration

Following surgical procedures, antibiotic prophylaxis is often administered for a short period of time, typically no longer than 24 hours. It is possible that the probability of developing an illness that is resistant to antibiotics may increase if preventive antibiotics are used for a lengthy period of time. In certain circumstances, such as when the patient is undergoing joint replacement surgery, the prophylaxis period may be prolonged for an extra 24 to 48 hours depending on the precise recommendations that are being followed and the risk factors that are associated with the patient [33,36].

Patient Risk Factors

There are a variety of patient-specific characteristics that might influence the antibiotics used, the administration schedule, and the length of treatment. Prophylaxis may need to be administered for a longer period of time to patients who are at a higher risk, such as those who have diabetes, obesity, or a weaker immune system [31-37].

Guidelines and Recommended Methods

The recommendations and best practices for antibiotic prophylaxis are often developed by surgical societies and infectious disease experts. Orthopedic surgeons are required to adhere to these guidelines and best practices. These guidelines, which are based on the most recent available information, have been developed with the intention of improving patient outcomes while simultaneously reducing the likelihood of antibiotic resistance [15-26].

The timing and duration of antibiotic prophylaxis are two of the most critical variables in lowering the incidence of SSIs following orthopedic surgery. During surgery, antibiotics must be timed for optimal therapeutic levels. On the other hand, reducing the length of time that antibiotic prophylaxis is taken can help lower the risk of antibiotic resistance. When deciding whether to start taking antibiotics and for how long, orthopedic surgeons and other healthcare practitioners need to stick to the established recommendations and take the patient's unique circumstances into account [31-37].

Effectiveness of antibiotic prophylaxis

Prophylactic Antibiotics Have Been Shown to Be Useful in the Prevention of Orthopedic Surgery Infections

In orthopedic surgery, the use of prophylactic antibiotics is crucial for preventing SSIs. These infections may result in severe repercussions, such as a failure of an implant, a protracted hospital stay, and greater medical costs. In this article, we examine the strong body of evidence supporting the value of antibiotic prophylaxis in orthopedic surgery [38].

Solid Historical Statistics

The use of antibiotics as a prophylactic measure during surgical operations extends back decades. There has been a discernible drop in the number of SSIs as a direct consequence of the use of preventive antibiotic regimens in orthopedic surgeries, as indicated by historical data [8-21]. These preliminary findings have established a foundation for its further use by laying the basis for it.

Instructions and Recommendations

Numerous organizations, including the AAOS, the CDC, and the WHO, have advocated for the use of prophylactic antibiotics prior to orthopedic surgery [21-24]. The consensus of this group of experts and the results of extensive research inform these recommendations, which highlight the usefulness of this technique.

Reduction of SSIs: Prophylactic antibiotics have been shown to reduce the incidence of SSIs after orthopedic surgery in a number of clinical trials and observational studies. A meta-analysis conducted by Classen et al. [8] indicated that the use of prophylactic antibiotics reduced the occurrence of SSIs by 81%. These results emphasize the significance of antibiotics as a strategy for preventing infections. Additionally, several studies have shown how crucial it is to choose the right antibiotics. Prophylactic antibiotics that have adequate tissue penetration and pathogen coverage have been shown to be extremely effective in earlier investigations [9-18]. A preoperative dose of antibiotics is typically ordered to be taken within a certain time window prior to incision. The timing of this medication is equally as important.

Economic efficiency: Antibiotics used as a preventative measure have been proven to be cost-effective, in addition to the clinical advantages they provide [36,37]. SSIs typically cause patients to be hospitalized for a significant amount of time and require further surgical operations. By avoiding these consequences, which are an important consideration in the present-day context of the healthcare industry, prophylactic antibiotics help to cut down on the expense of medical treatment.

Clinical outcomes: To improve a patient's overall quality of life is the overarching goal of orthopedic surgery [35,38]. The use of antibiotics as a preventative measure helps achieve this objective by lowering the likelihood of postoperative infections, which are known to be associated with incapacitating complications and unfavorable results. The use of antibiotic prophylaxis improves the patient's chances of making a full recovery.

Antibiotic resistance considerations: Even though the benefits of using antibiotics for preventative purposes are obvious, antibiotic stewardship is still very important [33-34,37-38]. Reduce the usage of antibiotics when they are not required, choose drugs appropriately, and adhere closely to dosage instructions. The possibility of antibiotic resistance, which is an increasing issue for the health of people all over the world, can be reduced with the aid of this technique.

According to the conclusion, the data that supports the usefulness of prophylactic antibiotics in orthopedic surgery is substantial and persuasive. Antibiotic prophylaxis has been proven, time and time again, to minimize the occurrence of SSIs, improve patient outcomes, and save healthcare costs. It is crucial that healthcare practitioners continue to adhere to established guidelines and practices responsible for antibiotic stewardship in order to guarantee the continuous efficacy of these interventions in orthopedic surgery. These recommendations and practices were developed to reduce the risk of antibiotic resistance.

Studies Showing a Reduction in SSIs With Antibiotic Prophylaxis in Orthopedic Surgery

Antibiotic prophylaxis' effect on postoperative infections in orthopedic surgery was studied by Classen et al. in the New England Journal of Medicine [8]. The study found that the incidence of infection at the surgical site was much diminished when prophylactic antibiotics were administered prior to orthopedic surgeries, emphasizing the need to give antibiotics at the right time before surgery. In their study, Bratzler and Houck outlined best practices for administering antibiotic prophylaxis during a variety of surgical procedures, including orthopedic surgery [9]. This places an emphasis on selecting the proper antibiotics, administering them at the appropriate time, and maintaining prophylaxis for the necessary amount of time to reduce the risk of SSI. This research by Sousa et al., while not a direct study on antibiotic prophylaxis, emphasizes the necessity of diagnosing and treating asymptomatic bacteriuria (ASB) before orthopedic surgeries [10]. Although this research was not a direct study of antibiotic prophylaxis. The treatment of ASB might be thought of as a sort of antibiotic prophylaxis in order to lessen the likelihood of SSI. Research conducted by de Beer and colleagues especially focused on total knee replacement procedures, and they discovered that antibiotic prophylaxis was helpful in lowering the number of SSIs [11]. This underscores how important it is to choose suitable antibiotics depending on the patterns of bacterial resistance that are found locally. Pamilo et al. conducted an analysis of SSIs that occurred following knee arthroplasty using data obtained from a joint replacement registry [12]. This highlights the risk factors for SSI and the relevance of infection prevention efforts, both of which provide support for the use of antibiotic prophylaxis in an indirect manner. In the research by Bryson et al., published in the Journal of Bone and Joint Surgery, we investigated the utility of antibiotic prophylaxis prior to elective foot and ankle surgery [4]. They determined that SSI rates dropped dramatically when the right preventive antibiotics were used. The question of whether antibiotic prophylaxis might reduce the risk of SSIs following total hip and knee arthroplasty was the topic of research conducted by Suratwala and colleagues [16]. The findings demonstrated a correlation between successful prophylaxis and fewer instances of SSI. In order to determine whether or not prophylactic antibiotics reduce the risk of SSI following orthopedic surgery, Giles et al. used data from the National Surgical Quality Improvement Program (NSQIP) [17]. The study found that the rate of SSIs dropped when prophylactic antibiotics were given before surgery. Tang et al. conducted a comprehensive review and meta-analysis to examine the efficacy of antibiotic prophylaxis in reducing the incidence of SSI in patients after spine surgery [18]. After completing the experiment, researchers found that prophylactic antibiotics helped reduce SSI rates. Hawn et al. looked at the optimal time of antibiotic prophylaxis for a variety of surgical procedures [13]. The incidence of SSIs was shown to be greatly reduced when antibiotics were administered at the ideal time (typically within 60 minutes before incision). Despite not concentrating explicitly on orthopedic surgery, Rodríguez-Caravaca et al.'s emphasis on the need for hospital-wide antibiotic control systems was noteworthy [6]. In a number of surgical settings, these programs can help ensure the best practices for antibiotic prophylaxis and reduce the frequency of SSI. This Mangram et al. guideline provides recommendations for the prevention of SSIs, including antibiotic prophylaxis [19]. It is widely acknowledged in surgical practice. The effect of antibiotic choice and timing on SSIs that developed after joint arthroplasty was examined in this research by Cojutti et al. [20]. They came to the conclusion that SSIs can be decreased by selecting the appropriate antibiotic and using it at the appropriate time.

Despite the fact that these studies showed that antibiotic prophylaxis can effectively reduce SSIs during orthopedic surgery, it is important to remember that prophylaxis effectiveness can vary depending on a variety of factors, including the kind of antibiotic used, when it is administered, the patient's characteristics, and how strictly infection control procedures are followed. Clinical standards and practices might alter over time, so it's critical to evaluate the most recent research and recommendations to get the most up-to-date information possible.

Discussion of the impact on surgical outcomes with regard to antibiotic prophylaxis usage

Antibiotic prophylaxis [36-38] has been the subject of a substantial amount of research because of the major influence it has on the results of surgical procedures. The following is a description of the fundamental aspects of this practice. Antibiotic prophylaxis is administered with the primary purpose of lowering the frequency of SSIs. One of the most common complications following surgery is an infection at the incision site. Extended hospital stays, higher medical bills, and even mortality have all been linked to these illnesses. The prevalence of SSIs may be greatly reduced with antibiotic prophylaxis, as has been proved time and time again in scientific studies.

The Antibiotics Selection 

Antibiotics used for prophylaxis must be chosen with great caution [39-40]. It needs to be based on a particular technique, the microorganisms that are often linked with surgery, and the antibiotic resistance patterns that are prevalent in the region. Antibiotics with a wide spectrum of activity are routinely prescribed for the treatment of a diverse range of possible infectious agents. On the other hand, overuse of antibiotics may potentially contribute to the development of antibiotic resistance. As a result, it is essential to strike a balance between the effectiveness of antibiotics and using them in a responsible manner.

When to Administer It and How to Do It

It is of the utmost importance to provide antibiotics at the most appropriate moment. In most cases, antibiotics have to be provided a short while before surgical incisions in order to guarantee that therapeutic doses are available in the tissues at the time that potential bacterial exposure takes place. The "golden hour" technique is absolutely necessary in order to achieve the highest possible level of preventative effectiveness [41].

The Length of Time 

In most cases, prophylactic antibiotics should be stopped within 24 hours following surgery [36-37,42]. This is because prolonged administration might raise the risk of antibiotic resistance without bringing any further advantages to the patient.

Factors of Potential Harm and the Selection of Patients 

In some surgical procedures, the use of antibiotic prophylaxis is not required [39]. Evaluation of individual patient risk factors, which may increase the incidence of SSI is required. These risk factors include diabetes, obesity, smoking, and immunosuppression, among others. Patients who are considered to be at high risk are the ones who are most likely to receive prophylaxis [36]. Even when used as a preventative measure, the usage of antibiotics has been shown to contribute to the evolution of microbes that are resistant to antibiotics. As a consequence of this, it is absolutely necessary to make careful use of antibiotics, stick to recommendations, and monitor local trends of resistance. Antibiotics are known to produce unintended consequences, the most common of which are allergic responses and gastrointestinal disorders. It is critical to reduce these risks as much as possible by choosing antibiotics that are appropriate and doing an assessment of the patient's sensitivities and intolerances.

Efficiency in Terms of Costs 

Antibiotic prophylaxis may result in a rise in short-term healthcare costs as a result of drug expenses; however, it typically results in a reduction in long-term healthcare costs by lowering the incidence of SSIs, which are known to lead to longer hospital stays and further operations [38].

In conclusion, antibiotic prophylaxis is an essential component of modern surgical practice since it considerably lowers the risk of SSIs, which is common and can lead to major complications after surgical procedures. However, it must be utilized prudently, with great attention given to the time of antibiotic administration, patient variables, and the antibiotic choice. Maintaining a healthy equilibrium between the advantages of infection prevention and the hazards of antibiotic resistance is a task that never goes away. Therefore, healthcare practitioners need to keep up with the latest research and guidelines in order to optimize the benefits of surgical procedures while simultaneously reducing any collateral damage.

Controversies and challenges

Explanation of Antibiotic Resistance and Its Implications

Resistance to antibiotics, drugs used to kill or slow the growth of bacteria and other microorganisms, is known as antibiotic resistance [43]. It's common practice to use antibiotics to treat a wide range of bacterial illnesses. This resistance develops as a result of the evolution of bacteria's defense mechanisms against antibiotics, which renders the medications useless [44,45]. Antibiotic resistance is a worldwide problem that impacts a variety of medical subfields, including orthopedic surgery. It is a threat to public health everywhere. In this article, we will discuss antibiotic resistance as well as the effects it has on orthopedic surgery.

Mechanisms Underlying Antimicrobial Resistance

Mutation: Bacteria have the ability to produce mutations in their genetic material that allow them to survive after being exposed to antibiotics [46,47].

Transfer of genes between different species: Bacteria are able to acquire resistance genes from other bacteria, even those of different species, through the processes of conjugation, transformation, and transduction [48].

Efflux pumps: Bacteria have the ability to get rid of antibiotics before they have the chance to do their job. Antibiotics may become useless because certain microbes develop enzymes that counteract their effects [49,50].

Consequences for Orthopedic Surgery

Antibiotics are routinely given in a preventative manner prior to orthopedic surgery in order to ward against the development of postoperative infections [51-54]. However, antibiotic resistance can lessen the effectiveness of these preventative measures, which is a problem. Orthopedic procedures including joint replacements and spinal fusions have the potential to introduce germs into the body, which can then result in postoperative infections [52,53]. Infections that develop after surgery can be challenging to treat if the germs causing them are resistant to drugs. Infections brought on by bacteria resistant to antibiotics can extend patients' stays in the hospital after undergoing orthopedic surgery, which can lead to an increase in both the expense of healthcare and the risk of problems [54]. Infections that are resistant to antibiotics might restrict the amount of drugs that are accessible for treatment, which can necessitate the use of treatments that are potentially more powerful, expensive, and harmful. When infections are unable to be treated successfully due to the resistance of antibiotics, there is an increased risk of mortality in instances that are very severe.

Mitigation and Prevention

Stewardship of antibiotics: Orthopedic surgeons and healthcare facilities should adhere to antibiotic stewardship programs in order to guarantee the proper use of antibiotics and to limit the risk of antibiotic resistance [55,56].

Containment of infections: In hospital operating rooms and other settings, as well as elsewhere in the healthcare industry, strict infection control procedures can help prevent the development of antibiotic-resistant bacteria as well as their dissemination. Patients must be made aware of the necessity of finishing the courses of antibiotic treatment that they have been prescribed in order to forestall the development of antibiotic resistance [57]. For a successful fight against antibiotic resistance, it is essential to continue the research and development of new medicines as well as alternative therapies.

In conclusion, orthopedic surgery and other areas of medicine are growing more concerned about the development of antibiotic resistance. This can result in postoperative infections that are more difficult to treat, increased healthcare expenses, and consequences that might possibly be deadly. Antibiotic resistance may be prevented and addressed via the employment of a multidimensional approach that includes responsible antibiotic use, infection control measures, and continuing research and development of novel medicines. This approach is necessary to assure the sustained effectiveness of orthopedic procedures and the safety of patients.

Discussion of potential risks associated with antibiotic prophylaxis and strategies to minimize

Antibiotic prophylaxis, often known as the use of antibiotics, to protect against infection, is a standard procedure in the medical field. It is utilized in a number of settings, such as right before dental or surgical treatments, as well as in specific medical problems [58,59]. Antibiotic prophylaxis has the potential to be very efficient in warding off infections; nevertheless, it also has the possibility of causing adverse consequences. In this part, we will review these risks and the measures that may be used to mitigate them.

Potential Dangers

Antibiotic resistance: The formation of bacteria that are resistant to antibiotic treatment can be caused by the inappropriate or excessive use of antibiotics. Microbes can acquire resistance to antibiotics when they are supplied when it is not necessary to do so, which makes it more difficult to treat illnesses in the future [60,61]. Antibiotics have been linked to a variety of allergic responses, ranging from relatively harmless rashes to potentially fatal anaphylaxis. The continued use of antibiotics is associated with a higher incidence of these adverse effects [62].

Disturbing the balance of the microbiome: Antibiotics have the potential to upset the delicate bacterial equilibrium that exists inside the body, which can result in gastrointestinal distress, fungal infections (such as candidiasis), and even possible long-term health problems due to the alteration of the microbiome. Infections that are resistant to antibiotics are also known as "superinfections" [63-65]. Antibiotics have been linked to an increased risk of opportunistic infections, particularly in immunocompromised people.

Strategies for Minimizing Dangers

Prophylactic antibiotic treatment should only be used when it provides more advantages than it poses a threat. When determining the likelihood of infection for each individual patient, it is important to consider all possible mitigation strategies, including improved sanitation and other preventative steps [66].

Antibiotics with a limited spectrum are optimal: Antibiotics with a narrow spectrum are optimal because they target the specific bacteria that are most likely to cause an illness [67]. This has the overall effect of reducing its impact on the microbiota and increasing the likelihood of resistance developing.

Effectiveness and recommended dosage: It is imperative that antibiotics be provided at the proper dose and for the shortest amount of time that is practicable [66-71]. It is possible for the likelihood of medication resistance as well as other adverse effects to grow with longer treatment regimens and greater dosages [68]. It is important for medical personnel to determine whether a patient is allergic to antibiotics and make a note of this information before providing antibiotics. It is possible for antibiotics to interact negatively with one another; nevertheless, having a thorough understanding of the patient's medical history can help in the prevention of serious allergic responses [69]. Patients must be made aware of the significance of strictly adhering to the antibiotic treatment plan that has been recommended for them, as well as the risks that are involved with failing to do so. In addition, patients should be urged to report any side effects as soon as they occur.

Reporting and surveillance: Healthcare institutions should set up reporting and surveillance systems to monitor antibiotic utilization and trends of resistance [70,71]. These data can help direct the choice of antibiotics and contribute to the identification of newly emerging resistance. Antibiotic stewardship programs should be implemented at hospitals and other healthcare institutions in order to encourage the responsible use of antibiotics, cut down on the number of improper prescriptions, and educate medical personnel on the hazards associated with antibiotic misuse. In addition to antibiotic prophylaxis, healthcare practitioners should make infection control measures a top priority to prevent the transmission of illnesses. These infection control methods include proper hand hygiene, aseptic procedures, and environmental cleanliness. The investigation of the use of vaccinations, improved surgical methods, and other infection control approaches can be used as potential replacements for antibiotics in the fight against infectious diseases.

Justification of antibiotic prophylaxis in orthopedic surgery

Antibiotic prophylaxis is warranted in orthopedic treatments for a variety of reasons, the most important of which are to preserve the safety of the patient and to improve the outcomes of the surgical procedure. The most important ones are as follows: Lessening the possibility of contracting an infection: During orthopedic treatments, it is possible for foreign items such as screws or joint prostheses to be placed in the patient. If the disease spreads, these implants might constitute a breeding ground for germs. Antibiotic prophylaxis reduces the number of germs present during surgery, which in turn reduces the likelihood that the surgical site will get infected. When left untreated, SSI can result in serious complications such as deep tissue infections, the formation of abscesses, and even sepsis in the worst-case scenarios. In extreme cases, the effects of these factors may lead to an extended stay in the hospital, further surgical procedures, a lifelong impairment, or even death. Antibiotic prophylaxis is a preventative measure that is used to avoid the potentially disastrous repercussions of the infection. Improving postoperative recovery infections can make the recovery process take longer, can slow wound healing, and can make pain worse. Antibiotic prophylaxis can help patients return to normal activities following surgery more quickly by reducing their risk of contracting infections.

Savings on Medical Expenses

The treatment of SSIs can be expensive since it often involves lengthy hospital stays, additional operations, and the need for long-term antibiotic treatments. Antibiotic prophylaxis, on the other hand, is an inexpensive choice since it has the potential to cut the total cost of orthopedic surgeries by a substantial amount.

Normative Phrases Taking Observation

Guidelines have been produced promoting antibiotic prophylaxis in certain orthopedic procedures by a number of healthcare organizations and regulatory authorities, including the WHO and the CDC. The professionals in the healthcare industry adhere to these principles in order to provide their patients with the highest possible level of care.

Antibiotic Resistance Prevention

Even in cases when surgery requires the administration of antibiotics, a strategy known as single-dose prophylaxis can help lower the probability that bacteria will develop resistance to antibiotics. The emergence of antibiotic-resistant bacteria is a significant threat to the general population since antibiotics can be misused or overused, both of which contribute to the problem. Antibiotics for prophylaxis could be chosen for a patient depending on the particular needs of that patient as well as any known allergies or sensitivities that the patient might have. This customization lessens the likelihood of patients experiencing adverse effects while also ensuring that they receive the required preventative care. As a consequence of this, antibiotic prophylaxis in orthopedic surgery is a tried-and-true strategy that aims to prevent SSIs, minimize complications, improve patient outcomes, and save costs associated with medical care. It is an essential component of the surgical care that is provided today, and the implementation of it is guided by guidelines that are based on evidence.

## Conclusions

Antibiotic prophylaxis, in conclusion, plays an essential part in the prevention of infections in a number of medical contexts; nevertheless, it must be provided with discretion and care in order to be effective. Antibiotic prophylaxis necessitates the utilization of a multifaceted strategy including not only patients but also healthcare practitioners and institutions, with a significant focus on antibiotic stewardship and infection control measures. This is necessary in order to reduce the potential adverse effects of antibiotic prophylaxis.
